# Application of Carbonic Nanosheets Based on Urea Precursors as Dispersive Solid Phase Extraction Adsorbent for Extraction of Methamphetamine from Urine Samples

**DOI:** 10.34172/apb.2021.071

**Published:** 2020-10-20

**Authors:** Arezou Taghvimi, Siavoush Dastmalchi, Yousef Javadzadeh

**Affiliations:** ^1^Biotechnology Research Centre, Tabriz University of Medical Science, Tabriz, Iran.; ^2^Faculty of Pharmacy, Tabriz University of Medical Sciences, Tabriz, Iran.; ^3^Faculty of Pharmacy, Near East University, POBOX:99138, Nicosia, North Cyprus, Mersin 10, Turkey.; ^4^Drug Applied Research Center, Tabriz University of Medical Sciences, Tabriz, Iran.

**Keywords:** Graphitic carbon nitride, Dispersive solid phase extraction, Methamphetamine, Urine

## Abstract

***Purpose:*** This paper established the application of synthesized graphitic carbon nitride nanosheets (GCNNs) as an influential dispersive solid phase extraction (DSPE) adsorbent in extracting methamphetamine from complicated urine media coupled with high performance liquid chromatography.

***Methods:*** The graphitic carbon nitride nanosheets (GCNNs) was synthesized easily and applied as adsorbent in the extraction process. The effective extraction parameters were investigated by one-parameter-at-a-time. Under optimized conditions the method was validated.

***Results:*** The calibration curve was plotted in the concentration range of 50-1500 ng/mL through the optimized conditions and the proposed method was validated. The method was used for the analysis of positive urine samples and showed satisfactory results with the average 99.7% relative recovery.

***Conclusion:*** The results persuade the capability of this novel method in analyzing of the positive urine samples in diverse clinical and forensic laboratories.

## Introduction

Methamphetamine is a highly potent central nervous system stimulant abused worldwide.^1,2^ Its side effects cause serious social problems, such as committing suicide, public disorder, and crimes.^3^ Methamphetamine, further, leads to mental and physical disorders in the subjects.^4,5^ Therefore, the introduction of novel analytical methods to discriminate methamphetamine is of great importance.^6,7^ Several sample preparation methods are applied in complex biological media due to complicated matrix and deficient dosages of the analytes in them. Several sample preparation methods are applied in complex biological medias due to complicated matrix and deficient dosages of the analytes in the media. Urine is one of the most applied biological fluids due to accessibility in high volumes and being non-invasive media.^8^ The complexity of the urine requires a sample preparation step before analysis. Liquid-liquid extraction (LLE) and solid phase extraction (SPE) are regularly applied as sample preparation methods. The application of the LLE method is limited due to being laborites, time-consuming, as well as high consumption of toxic organic solvents. Probable frangibility, clogging risk of the SPE cartridges, and solvent peculation through solid-phase particles are SPE disadvantages.^9^ In order to cover the mentioned problems, the dispersive solid phase extraction (DSPE) method is selected due to the advantages of high extraction efficiency, lower desorption organic solvents, and favorable preconcentration factor.^7^ There are different methods that have been applied for methamphetamine extraction and determination of methamphetamine from urine samples. A paper reported electro-enhanced single-drop microextraction (EE-SDME) coupled with gas chromatography for amphetamine and methamphetamine extraction from urine media.^10^ In another work, 1-Octyl-3-methylimidazolium hexafluorophosphate as an ionic liquid, along with methanol was used as microextraction solvent in ionic liquid-based DLLME (IL-DLLME) combined with high performance liquid chromatography ultra violet detector method for effective extraction of MET from urine.^11^ Moreover, head space developed solid phase micro extraction (SPME) gas chromatography mass spectrometry method was applied for amphetamines extraction from urine while the PDMS fiber was derivatized byexposing the fiber to trifluoroacetic anhydride after the extraction. The derivatized analytes were desorbed in gas chromatography.^12^ Graphene oxide–Fe_3_O_4_ nanocomposite was used as magnetic solid phase extraction (MSPE) adsorbent in simultaneous extraction of amphetamines from urine coupled with ultrahigh performance liquid chromatography-tandem mass spectrometry (UHPLC-MS/MS).^13-15^ Zeolite imidazole framework (ZIF-8) was applied as DSPE adsorbent in extraction of methamphetamine from urine. The method is fast and cost-effective with high recovery rate.^7^ The physical and chemical structure of the adsorbent discloses an influential function in achieving high extraction recovery of the analyte. The presence of nanostructure adsorbents in the DSPE method dramatically increases the analyte and the adsorbent interaction, leading to high extraction efficiencies.^10,11^ Graphitic carbon nitride nanosheets (GCNNs) are a novel class of carbon-based materials with unique properties.^16^ GCNNs present an extended delocalized π electron system, persuading a well π-interaction towards compounds with aromatic benzene ring in their structure. A strong covalence C-N band exists in each layer consisting of tri-s-triazine connected via tertiary amines. The Vander Waals interaction presents between layers. Two sides of the polyaromatic scaffold of the planar sheets are accessible for adsorption of the analyte from the media.^17^ GCNNs are commonly synthesized by facial and cost-effective pyrolysis of precursors containing carbon and nitrogen, such as melamine, thiourea, urea, cyanamide, and dicyandiamide without applying organic solvents.^18^ The remarkable structural properties of the GCNNs encourage its application in different fields, including hydrogen storage, electro-generated chemiluminescence, photocatalysis, lithium-ion battery.^19-23^ Beside mentioned analytical applications of GCNNs, this material is a great candidate as adsorbent in different sample preparation methods such as SPE and MSPE.^23,24^

In this study, GCNNs are synthesized by simple pyrolysis of urea, and after full characterization, it is employed in the DSPE process of methamphetamine extraction from urine media. The introduced method might operate in various laboratories for methamphetamine assay.

## Materials and Methods

### Materials

Methamphetamine hydrochloride stock solution 1000 µg/mL in methanol was obtained from Sigma-Aldrich (USA). Urea, KH_2_PO_4,_ HCl, and NaOH were provided from Merck Chemicals (Darmstadt, Germany). Acetone, methanol, and acetonitrile (HPLC grade) were purchased from Merck Chemicals (Darmstadt, Germany). Ultrapure water was obtained from the Milli-Q water system (Darmstadt, Germany).

### Instruments

Infrared spectroscopy was recorded by the Tensor 27 FTIR (Bruker, Germany). Scanning electron microscopy (SEM) (MIRA3 FEG–SEM, Tescan, the Czech Republic) was used to report the size of the nanoparticles. Zeta potential measurements were carried out using a Zetasizer (Nanotrac Wave, Microtrac, Germany). Powder X-ray diffraction patterns (XRD) were reported on the D5000 (Siemens, Germany) instrument. The HPLC analysis was conducted by Knauer (Germany) system equipped with a UV–visible detector (K-2600, Knauer, Germany). Analytical C_18_ column (5 µm particle diameter, 4.6 mm i.­ d. × 25 cm) (Knauer, Germany) was applied for separation at room temperature. The mobile phase was composed of an acetonitrile-phosphate buffer consisting of 15:85 (V/V), (10 mM, pH=3.5) at the flow rate of 1 mL/min.

### Biological urine sample preparation

Three healthy volunteers were involved in the research. The urine samples were collected and kept at 4℃. Positive subjects samples were provided from the MAHAN therapy center (Tabriz, Iran). The investigation was started by spiking 0.1 µg/mL methamphetamine to 4 mL of the healthy volunteer urine. The pH of the sample was alkalized to 10, resulting in insoluble solid particle precipitation. Direct injection mode of urine requires a previous sample treatment in order to achieve an efficient extraction of the analytes while maintaining the good performance of the extraction. Centrifugation is necessary to precipitate urinary sediments. Organized urine sediment consists of biological elements such as leukocytes, erythrocytes, epithelial cells, casts, bacteria, fungi, parasites and sperm. Unorganized urine sediment contains crystals of various salts, for instance oxalate, phosphate, urate, and amorphous salts. The insoluble particles were separated by centrifugation at 5500 rpm for 15 min (universal 320, Pole Ideal Tajhiz Co., Iran).^8^ The supernatant was moved to another container and preserved at 4℃ for the next extraction steps.

### Extraction procedure

Four milliliters of spiked urine matrix with the concentration of 0.1 µg/mL methamphetamine was selected, and 10 mg of the GCNNs adsorbent was added into the sample. Then, the sample was sonicated (Farasout, Iran) for 4 minutes to provide the maximum interaction between the analyte and the adsorbent through π-π interaction. Next, it was centrifuged, and the supernatant was dropped away. 400 µL of acetone was added as the desorption solvent and sonicated for 5 minutes later. The resulted solution was centrifuged once more, and 20 µL of the supernatant was injected into the HPLC-UV system.

### GCNNs synthesis process

The synthesis of GCNNs was performed by a facial and one-step process. For this purpose, 20 g of urea was pyrolyzed at 550°C for 4 hours by a heating rate of 4°C min^-1^. Later, the obtained yellowish powder was washed with deionized water three times, and then it was dried at 60°C. The resulted powder was employed as the adsorbent in the experiment.^18^ The resulted powder was employed as the adsorbent in the experiment.

## Results and Discussion

### Characterization of GCNNs

**Figure 1:**
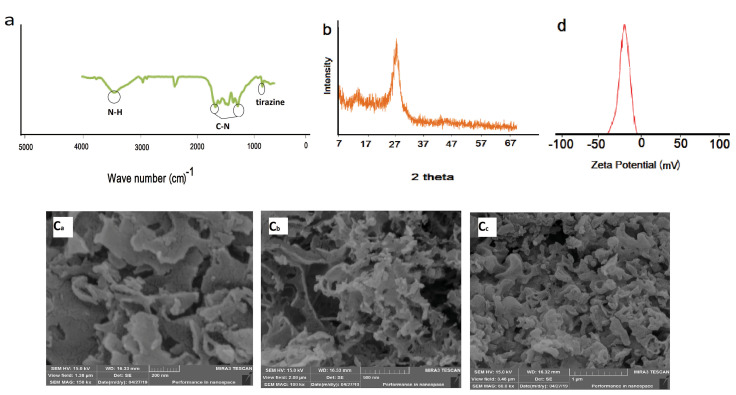
(a)Presents the Fourier transform spectroscopy (FTIR) of the GCNNs, (b) X-ray diffraction (XRD) pattern of the synthesized GCNNs, (c) _(1,2 and 3)_) Scanning electron microscopy (SEM) image of GCNNs with three different magnifications (200, 500 nm and 1 µm) and (d) Zeta potential value.

Figure 1a shows the FTIR spectroscopy of GCNNs. Peaks at 809 cm^-1^ and 1245-1640 cm^-1^ are related to the breathing mode of triazine and stretching mode of C-N heterocycles, respectively. A broad peak at 3000-3500 cm^-1^ is associated with the vibration of the N-H band. The obtained results are in good accordance with previously published papers.^25,26^ The XRD of the synthesized GCNNs is shown in Figure 1b. A peak at 2Ɵ = 12.6° is correspondence to the in-planar structural packing motif of the GCNNs common structure. A sharp peak at 2Ɵ = 27.08° is related to the stacking of the conjugated aromatic system as in the graphite. The results are in good agreement with the literature.^18,27^ The morphological study of GCNNs is conducted by scanning electron microscopy (SEM). The SEM images show a layered structure of GCNNs without any amorphous structure with an estimated average size of about 22 nm (Figure 1c_1_). The zeta potential value of the 1 mg/mL GCNNs dispersion is – 25.2 mV (Figure 1d). The presence of negative charges of the large delocalized π electron system on GCNNs provides well-dispersion of them in the matrix. This facet improves the analyte and GCNNs interactions, persuading high extraction efficiencies.

### Comparison of graphene and GCNNs carbon-based adsorbent extraction efficiency

**Figure 2:**
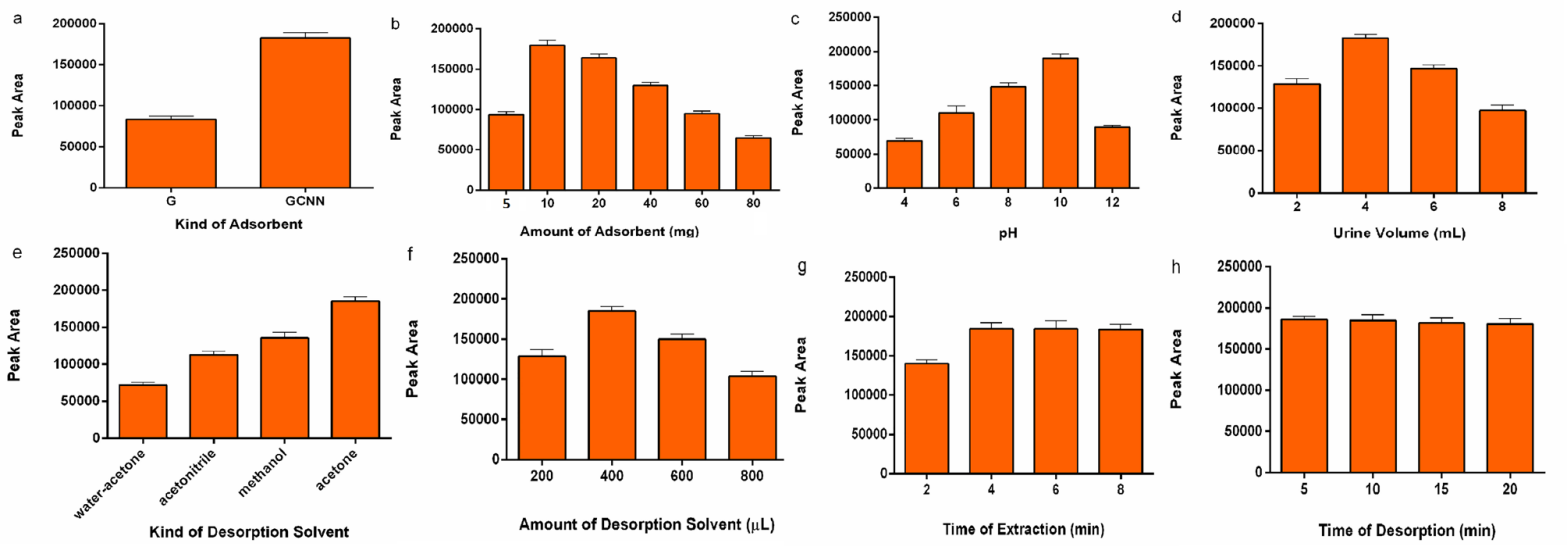
Optimization of the effective extraction parameters by proposed DSPE method in presence of 0.1 µg/mL spiked methamphetamine in urine media.

Graphene is a famous carbon-based material with different application namely used as adsorbent in sample preparation methods. Graphene has been applied as a coating material in SPME method for efficient extraction of pyrethroid pesticides, organochlorine pesticides, phenols from water and soil samples.^24,28,29^ As GCNNs show a similar chemical structure to graphene (G) with a slight difference by presenting C-N bonds instead of C-C in the nanosheet as a tri-s-triazine unit, a comparison of extraction efficiency between the G and GCNNs is conducted in spiked 0.1 µg/mL methamphetamine in urine matrix. Results demonstrate that GCNNs have two-fold higher extraction efficiency to methamphetamine (Figure 2a).

### Amount of adsorbent

The optimum amount of the adsorbent affects the extraction efficiency as it may provide the highest interaction surface area among the GCNNs and the analyte. For this purpose, various amounts of the adsorbent in the range of 5-80 mg are studied. The results reveal that when 10 mg of the adsorbent is employed, the maximum peak area is obtained. Gradual enhancement of the adsorbent amount diminishes the extraction efficiency. This could be due to the stacking of the nano GCNNs particles, which prevents the effective π-π interaction of analyte and GCNNs surface area (Figure 2b).

### Effective extraction pH, urine volume and ionic strength

The value of pH in the urine matrix may increase or decrease the extraction efficiency by changing the charges on the adsorbent surface and the matrix. This state may differentiate the influential interactions of the GCNNs and methamphetamine. The pH value of urine is investigated in a variety of 4-12. In the pH value of 4, due to the presence of high positive ions in the urine media that decreases the amount of effective negative charges on the GCNNs, the interaction of the GCNNs and positively charged analyte declines, as shown in Figure 2c. By gently increasing the pH up to 10, the amount of the negative charges increases, leading to an enhancement of the extraction efficiency. It is clear that the pK_a_ value of the methamphetamine is 10.1,^30^ and by a gradual increase of the pH to 12, the analyte changes to the neutral form, showing no tendency towards negatively charged adsorbent, thus declining extraction. Both π-π and electrostatic interactions (negative charges due to presence of extended π-π electron system of the GCNNs and phenolic cycle of the methamphetamine on one hand and positive charges of the hydrochloride form of the analyte on the other hand) are responsible for analyte extraction. Variation in the pH value of the urine media, makes electrostatic interactions dominate in the pH value of 4-10. π-π interactions are constant in all pH values. In the pH value of 12 as the analyte is neutral, just π-π interactions are responsible for analyte extraction. Urine volume may affect the extraction efficiency. Different urine amount of 2-8 mL are studied (Figure 2d). The results show that enhancement of the urine volume from 2 to 4 helps well-dispersion of the GCNNs in the urine, resulting in the effective analyte and GCNNs interaction. The increase of the urine volume up to 8 reveals a decrease in the peak areas. This phenomenon might be related to the dissociation of the GCNNs nanoparticles that could reduce the influential interaction. The ionic strength of the media is studied in the 0-5 % (W/V) NaCl as well. No obvious shift is seen in extraction efficiency; therefore, the procedure is continued without ionic strength adjustment.

### Optimum desorption organic solvent and its volume

The desorption solvent is an essential parameter in obtaining maximum extraction efficiency. Therefore, different solvents, including acetone, H_2_O-acetone (50:50), methanol, and acetonitrile, were examined (Figure 2e). According to the results, acetone presented maximum extraction efficiency due to the establishment of suitable interaction polarity between the adsorbent structure and the analyte. Next, the volume of the acetone was studied in the 200-800 µL range (Figure 2f). The data showed that the highest efficiency was achieved in 400 µL of the acetone, and a further increase of the acetone volume decreased the extraction efficiency due to analyte dilution.

### Extraction and desorption sonication time

In the equilibrium-based methods, the time of reaching the equilibrium is critical, which could affect the maximum interaction between the methamphetamine and the GCNNs. As the extraction is an equilibrium-based method, the optimization of the optimum extraction and desorption time is indispensable. The extraction time is investigated in the range of 2-8 min sonication. As can be seen in Figure 2g, the extraction efficiency increases during 2-4 minutes; however, by continuous increase of the sonication time there is no significant change in the extraction efficiency. This experiment is studied for desorption time covered in 5-20 minutes (Figure 2h). It is concluded that 5 minutes is enough for establishing the desorption equilibrium.

### Adsorbent capacity and reusability

The adsorbent capacity for synthesized GCNNs is obtained according to the following equation:


$${\text{Q}}_{\text{e}}=\left(\frac{({\text{C}}_{0-}{\text{C}}_{\text{e}})\text{V}}{\text{M}}\right)\times 100$$

**Figure 3:**
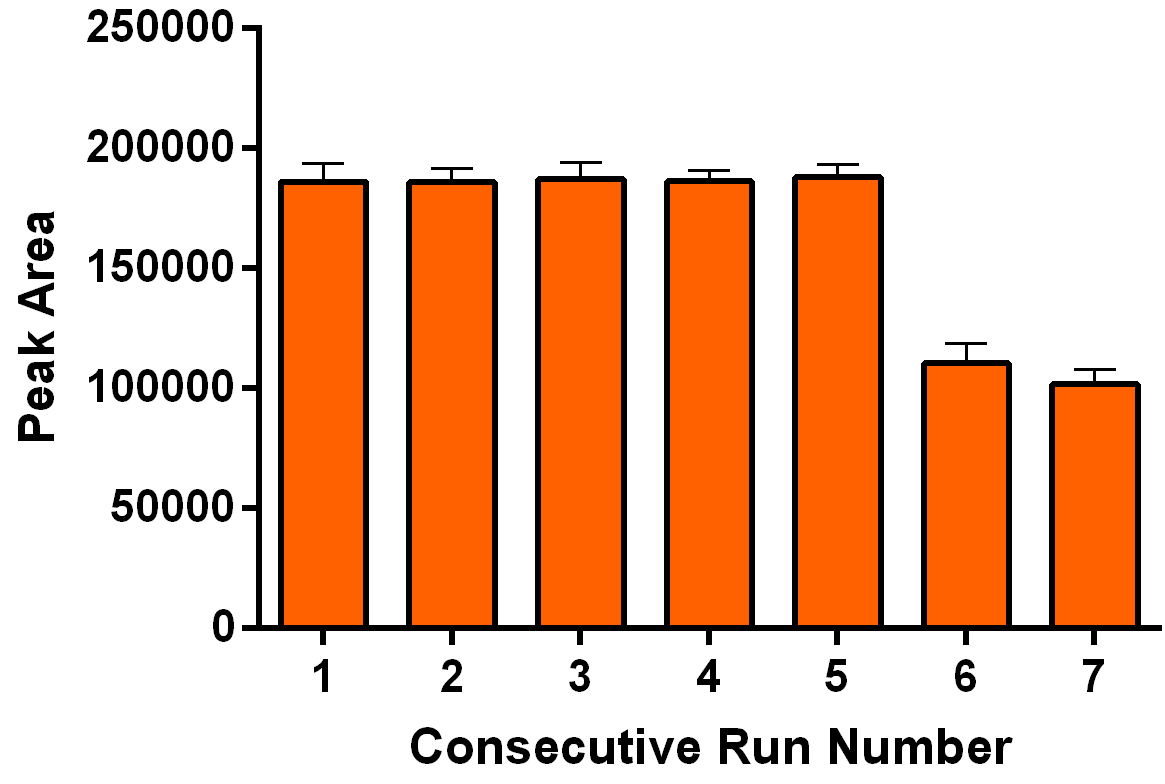
Reusability study of the synthesized GCNNs adsorbent under optimized extraction parameters in presence of 0.1 µg/mL methamphetamine in urine matrix.

Where C_0_ and C_e_ are the initial and equilibrium concentrations of methamphetamine in the solution (µg/mL), respectively. V is the urine volume (mL), and M is the adsorbent amount (g).^31^ The adsorption capacity is as 0.7 mg/g. The reusability of the GCNNs is studied, covering the optimized conditions (Figure 3). The outcomes reveal that extraction efficiency does not change during five times repetition (*P* < 0.05).

### Method validation

**Figure 4:**
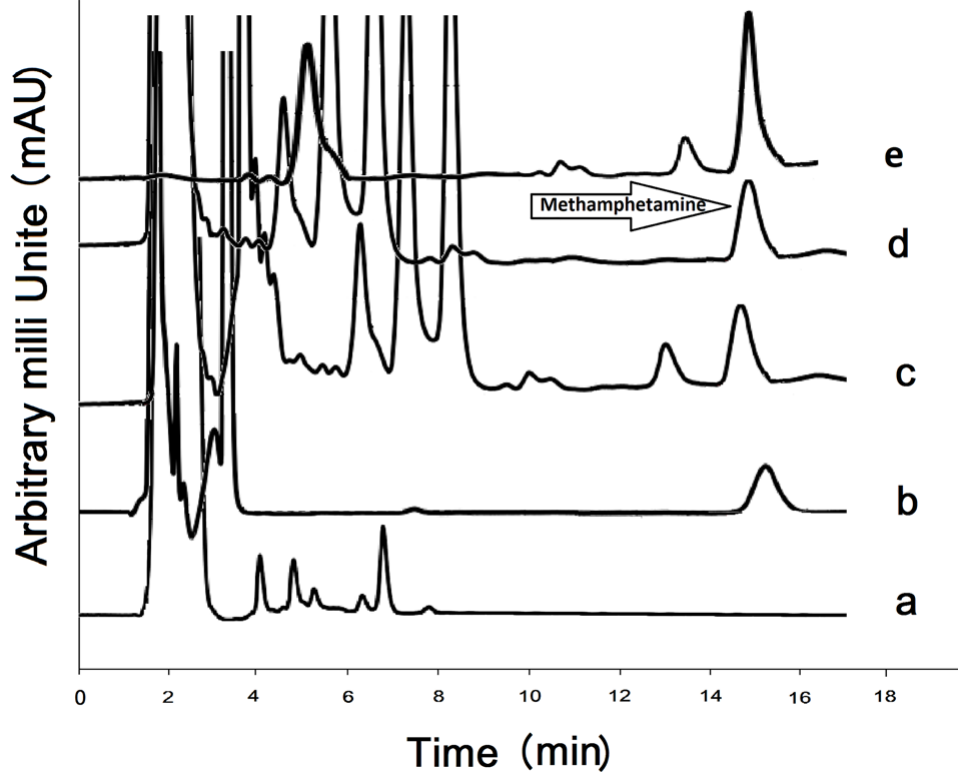
The calibration curve for the proposed DSPE method.

Some of analytical characteristic of the introduced method for methamphetamine determination such as limit of detection (LOD), coefficient of determination (r^2^), limit of quantification (LOQ), linear range, and relative standard deviation (RSD) were obtained as 14 ng/mL, 0.9934, 45.80 ng/mL, 50-1500 ng/mL and 5%, respectively. The data obtained through the calibration curve under optimized conditions for five concentrations repeated three times (Figure 4). The LOD and LOQ were calculated based on Signal-to-Noise (S/N) approach: Calculation of the S/N ratio is done by comparing signal intensity of known low concentrations of methamphetamine with those of noise (methamphetamine spiked sample were prepared under DSPE). A signal-to-noise ratio 3:1 is generally considered acceptable for estimating the detection limit. Determination of the LOQ is performed as described for LOD but S/N ratio of 10:1. RSD for (inter-day and intra-day experiments) was calculated as follows: The mean value of obtained extracted peak area by DSPE method from calibration curve (0.1, 0.5 and 1.5 µg/mL) was calculated. The proportion of SD to mean value of obtained peak area was calculated and the result was multiplied to 100. The absolute recovery (recovery percent) is the percent amount of methamphetamine recovered from urine matrix (extracted sample) versus the standard (unextracted). The recovery was calculated as 99.40 for the method presented in Table 1 presenting a comparison between the introduced extraction process with other commonly used methods.^32-34^ The results disclose that the adsorbent synthesis is very fast, facial and cost-effective than other methods mentioned in Table 2. In another words, handling of hollow fibers, application of electro membranes in extraction process or derivatization steps are slightly time consuming and not easy procedures for the operator. Moreover, use of commercial fibers or some chemical reagents such as ionic liquids may not be cost-effective in designing a novel extraction method. The instrumental restriction of the UV detector leads to obtaining slightly higher LODs in comparison to gas chromatography or mass spectroscopy. Table 2 is the analytical accuracy and precision of the developed method across the calibration curve. Enrichment factor (EF) was calculated as follows:


$$EF=\;\frac{\;AUC_{0.1\;\frac{\mu g}{mL}\;drug\;in\;urine}}{AUC_{0.1\;\frac{\mu g}{mL}\;STD}}$$

**Table 1:** Comparison of the introduced DSPE method with common existing methods

**Method**	**LOD** ^a^ **(ng/mL)**	**Time of Extraction** **(min)**	**Recovery** **(%)**	**RSD** ^i^ **(%)**	**Linear range** **(ng/mL)**	**Benefits /drawbacks of methods**	**References**
GO-EME-GC-FID^b^	2.40	20	95-98.50	5-6.60	8-800	Not easily handled	^32^
OCD-GC-MS^c^	250	-	102	7.70	500-5000	Derivitization step	^33^
SPME-GC-FID^d^	30	15	20-38.1	-	129-3876	Fragle, expensive	^34^
DSPE-HPLC-UV^e^	10	5	99.83	4.50	50-2500	Diferent expensive chemicals	^7^
EE-SDME-GC-FID^f^	0.14	40	82.70-90.50	8.9-12.8	1-2000	Difficult to handle	^10^
IL-DLLME-HPLC-UV^g^	1.7	16	-	5.20	10-1000	Toxic solvents	^11^
SPME-GC-MS	3	10	75.22-91.10	-	50-1000	Fragle, expensive	^12^
MSPE-UPLC-MS/MS^h^	0.02	15	92.23	5.50	0.05-1000	Lowr LOD	^13^
DSPE-HPLC-UV	14	4	99.40	5	50-1500	Slightly high LOD	This study

^a^ Limit of detection S/N=3; ^b^Graphene oxide assisted electromembrane-Gas chromatography-Flame ionization detector; ^c^ On column derivatization- Gas chromatography-Mass spectrometry; ^d^ Solid phase micro extraction- Gas chromatography-Flame ionization detector;^e^Dispersive solid phase extraction-High performance liquid chromatography-Ultraviolet; ^f^Electro-enhanced single-drop microextraction-Gas chromatography-Flame ionization detector; ^g^Ionic liquid dispersive liquid-liquid microextraction-High performance liquid chromatography-Ultraviolet; ^h^Magnetic solid phase extraction-ultrahigh performance liquid chromatography-tandem mass spectrometry; ^I^Relative standard deviation.

**Table 2:** Analytical precision and accuracy

**Analyte Concentration (µg/mL)**	**Intra-day (n=3)**			**Inter-day (n=3)**		
	**Recovery (%)**	**Precision RSD (%)** ^a^	**Accuracy (bias)**	**Recovery (%)**	**Precision RSD (%)**	**Accuracy (bias)**
0.1	99.86	4.7	1.4	99.56	3.2	1.1
0.5	99.32	4.9	0.9	98.62	5.6	1.3
1.5	98.63	3.8	1.2	99.73	4.9	0.9

^a^Relative standard deviation.

The EF was obtained as 112.

### Analysis of real samples

**Figure 5:**
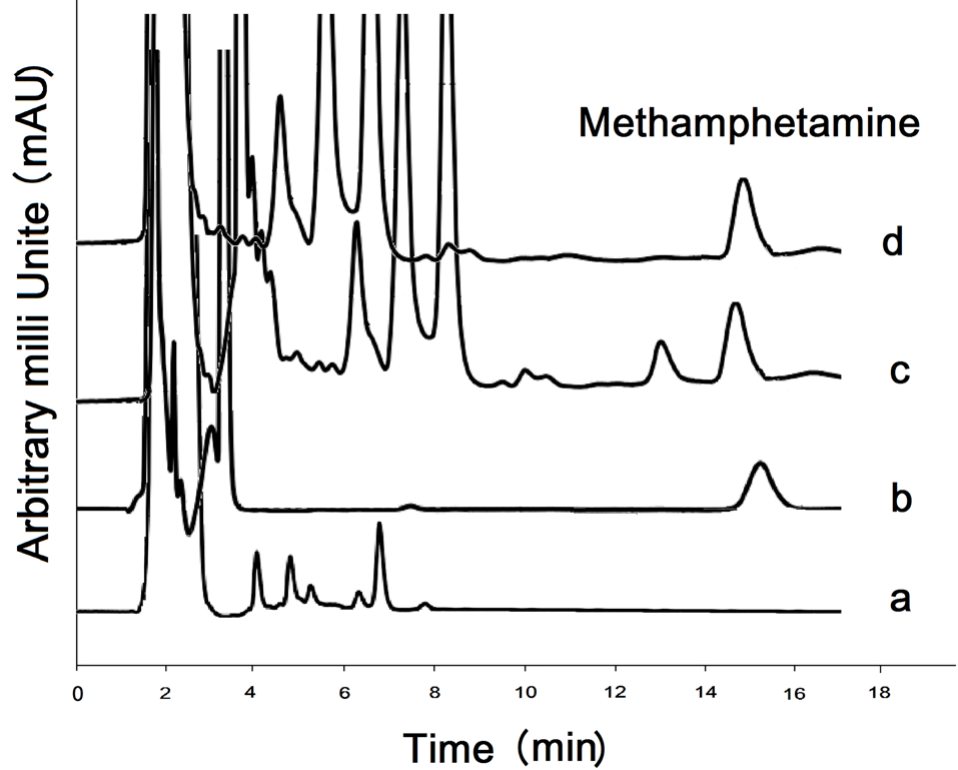
(a) Chromatograms of the blank and (b) spiked urine sample with 0.1 µg/mL methamphetamine, (c) chromatograms of positive urine sample and (d) selectivity study of the proposed method in the simultaneous presence of 0.1 µg/mL methamphetamine, amphetamine, ephedrine and pseudoephedrine in urine media, (e) Chromatogram related to real sample spiked with 0.1 μg/mL methamphetamine standard solution.

Blank and 0.1 µg/mL methamphetamine spiked urine sample chromatograms are illustrated in Figure 5a and b, respectively. Positive urine samples are analyzed by the GCNNs adsorbent shown in Figure 5c. Chromatogram related to real sample spiked with 0.1 μg/mL methamphetamine standard solution is presented in Figure 5e. Table 3 shows the results of applied proposed method in analysis of some real urine samples.

**Table 3:** Application of the proposed method in analysis of some real urine samples

**Real urine samples**	**Methamphetamine concentration (µg/mL)**	**Recovery (%)**	**Recovery (%) Added** **Concentrations to Real Samples (µg/mL)**		
			**0.1**	**0.5**	**1.5**
Subject 1	1.45	99.80	99.66	99.46	99.56
Subject 2	1.23	99.92	99.54	99.73	99.63
Subject 3	1.29	99.30	99.32	99.69	99.35

### Selectivity, matrix effect and robustness of the method

The selectivity of the method is achieved by simultaneous spiking of 0.1 µg/mL methamphetamine metabolites such as amphetamine and also some other drugs, including ephedrine and pseudoephedrine, in urine media, and the extraction process is carried out under optimized conditions. According to Figure 5d, no meaningful peak is seen in the analysis time. Moreover, the matrix effect is achieved by evaluating the degree of enhancement or reducing methamphetamine peak area made by the urine matrix as follow:


$$Matrix\;Effect\;\left(\%\right)=\frac{B}{A}\times 100$$

Where A is the external solution peak area, and B is the post-extraction sample peak area. The matrix effect is 106%, conforming to a minor matrix effect. Stability of the proposed method was studied as robustness parameter reported in Table 4.

**Table 4:** Robustness study of the proposed method by one-parameter-at-a-time method

**Variable**	**Variations**	**Retention Time (min)**	**RSD (%)**
Buffer pH	3.48	15.52	1.30
	3.5	15.10	1.48
	3.52	14.92	1.28
Buffer Concentration (mM)	8	14.29	0.73
	10	15.10	1.36
	12	14.72	1.26
Time of Extraction (min)	6	14.90	0.95
	4	15.10	1.34
	2	14.80	1.11

## Conclusion

The paper presents a novel sample preparation method using GCNNs as the DSPE adsorbent for the effective extraction of methamphetamine from urine matrix. The method showed high linear range with short extraction time. In addition, the method showed good precision, accuracy and robustness for methamphetamine detection. Methamphetamine could be detected without any significant interference effects of metabolites. Moreover, the facial synthesis process, simple precursors, and unique chemical properties of GCNNs encourage examines to apply them in various benzene ring drug extraction. The reasonable recovery results suggest the proposed method as an efficient tool in determining methamphetamine from urine in different clinical and forensic laboratories.

## Ethical Issues

All procedures performed in studies involving human participants were in accordance with the ethical standards of the institutional and/or national research committee and with the

1964 Helsinki declaration and its later amendments or comparable ethical standards.

## Conﬂict of Interest

The authors declare that they have no conﬂict of interest.

## Acknowledgments

The authors greatly appreciate the biotechnology research Centre of Tabriz University of Medical Science and Iran National Science Foundation for supporting this project. (Grant number: 96009553)
